# Variations in high density cholesterol levels based on apolipoprotein E variant and exercise type

**DOI:** 10.3389/fgene.2023.1136483

**Published:** 2023-06-14

**Authors:** Huan-Cheng Chang, Oswald Ndi Nfor, Chien-Chang Ho, Pei-Hsin Chen, Yung-Po Liaw

**Affiliations:** ^1^Division of Family Medicine, Department of Community Medicine, Landseed International Hospital, Taoyuan City, Taiwan; ^2^Department of Health Business Management Administration, Hungkuang University, Taichung City, Taiwan; ^3^Department of Public Health and Institute of Public Health, Chung Shan Medical University, Taichung City, Taiwan; ^4^Department of Physical Education, Fu Jen Catholic University, New Taipei, Taiwan; ^5^ Research and Development Center for Physical Education, Health, and Information Technology, Fu Jen Catholic University, New Taipei, Taiwan; ^6^Department of Medical Imaging, Chung Shan Medical University Hospital, Taichung City, Taiwan; ^7^ Institute of Medicine, Chung Shan Medical University, Taichung City, Taiwan

**Keywords:** exercise, HDL cholesterol, genetic predisposition, cardiometabolic factors, epidemiology

## Abstract

In various cross-sectional and longitudinal studies, exercise has been associated with cardiometabolic outcomes, including high-density lipoprotein (HDL) cholesterol. Exercise-induced changes in HDL cholesterol seem to be affected by genetic polymorphisms. In this study, we examined whether variant *APOE* rs7412 is involved in the association between HDL cholesterol and exercise. From adults assessed in Taiwan Biobank (TWB) between 2008 and 2019, we analyzed data from 57,638 normolipidemic subjects. To examine the association between exercise, *APOE* rs7412, and HDL cholesterol, a multiple linear regression model was used. A higher HDL was associated with both aerobic exercise (regression coefficient [mg/dL] beta- (β), 1.112; 95% confidence interval (CI); 0.903–1.322) and resistance exercise (β, 2.530; 95% CI, 2.093–2.966). In comparison with the *APOE* rs7412-CC genotype, the β was 2.589 (95% CI, 2.329–2.848) among those with the CT + TT genotype. Compared to adults who had the CC genotype and did not exercise (the CC/no exercise group), the β-coefficient determined for the different genotype and exercise groups was 1.135 (95% CI, 0.911–1.359) for the CC genotype and aerobic exercise group, 2.753 (95% CI, 2.283–3.322) for the CC genotype and resistance exercise group, 2.705 (95% CI, 2.390–3.020) for the CT + TT genotype and no exercise group, 3.682 (95% CI, 3.218–4.146) for the CT + TT genotype and aerobic exercise group, and 3.855 (95% CI, 2.727–4.982) for the CT + TT genotype and resistance exercise group, respectively. This study demonstrates that self-reported aerobic and resistance exercise both raised HDL levels, yet resistance exercise was associated with a greater increase, particularly among Taiwanese subjects carrying the *APOE* rs7412-CT+TT genotype.

## 1 Introduction

There is evidence that high HDL cholesterol plays a vital role in predicting cardiovascular events, and can be used as a therapeutic target in disease management (Barter et al., 2007). Previous studies have indicated that an increase in HDL cholesterol by just 1 mg/dL may reduce CHD risk by approximately 2%–3% (Sharrett et al., 2001; Ashen and Blumenthal, 2005). Research efforts have recently been focused on understanding the various physical activities associated with HDL cholesterol (Woudberg et al., 2018).

Exercise plays an instrumental role in managing lipoprotein levels associated with heart diseases (Stefanick et al., 1998). Numerous cross-sectional and longitudinal studies have demonstrated an association between cardiometabolic outcomes, such as HDL cholesterol and physical activity (Pattyn et al., 2013; Lin et al., 2015; Lemes et al., 2018). In a previous study, an 8-week aerobic exercise program was associated with an increase in HDL cholesterol levels, from 47.5 mg/dL to 52.5 mg/dL (Vazzana et al., 2013). Meanwhile, findings from another study (Lemes et al., 2016) have demonstrated that exercise, whether aerobic or resistance, does not result in clinically significant changes in HDL cholesterol.

The concentration of HDL cholesterol in our body reflects a combination of several factors, including environmental and lifestyle factors, as well as genetic factors, which account for approximately 50% of the changes (Heller et al., 1993; Cuchel and Rader, 2003). According to a previous review (Brunham and Hayden, 2015), about 62%–77% of the variance in HDL-C levels has been attributed to genetic factors even though the biological roles of the genes involved are still to be fully understood. Some of the genes linked to HDL cholesterol include the apolipoprotein E (APOE), apolipoprotein A-I (apoA-I), ATP binding cassette transporter, sub-family A, member 1 (ABCA1), lecithin cholesterol acyltransferase (LCAT), Cholesterol ester transfer protein (CETP), lipoprotein lipase (encoded by the LPL gene), hepatic lipase (encoded by LIPC), endothelial lipase (encoded by LIPG), phospholipid transfer protein (PLTP), the scavenger receptor class B member I (SR-BI, encoded by the SCARB1 gene), and others (Brunham and Hayden, 2015). Several variants of these genes have been associated with HDL cholesterol (Haase et al., 2010; Haase et al., 2012; Voight et al., 2012).

An important protein component of HDL cholesterol is *APOE* which is secreted by the liver and is involved in the hepatic uptake of triglyceride-rich lipoprotein. *APOE* rs7412 is one of the variants known to influence plasma lipid levels and other metabolic disorders (Bennet et al., 2007; Phillips, 2014). As far as we are aware, its influence on lipid metabolism has been widely studied, with the majority of studies focusing on low-density lipoprotein cholesterol (LDL-C) levels. Recently, Taiwanese researchers identified this variant as one of the most potent genetic factors influencing HDL cholesterol levels in Taiwan (Yeh et al., 2022). To gain a deeper understanding of this variant, we investigated whether its association with HDL cholesterol is related to exercise, which has been shown to improve lipoprotein levels and cholesterol efflux capacity (Stanton et al., 2022).

## 2 Materials and methods

### 2.1 Subjects

Data were available for 132,720 subjects within the TWB. These individuals were between the ages of 30 and 70 years old without a previous diagnosis of cancer. Their assessment in biobank centers took place between 2008 and 2019. Individuals participating in TWB are normally required to sign written informed consent before being assessed. Among the total subjects, 73,196 had genetic data. Our analysis, however, excluded those with hyperlipidemia (n = 5,496), as well as those with incomplete or missing data (n = 10,062). The final analysis models included 57,638 subjects. Each individual signed a consent form before undergoing assessments at biobank centers across the country. This study was approved by the Institutional Review Board of Lanseed International Hospital (IRB-206-B1).

### 2.2 Study variables and exercise program

Sociodemographic (age, sex, ethnicity), lifestyle (exercise, alcohol consumption, cigarette smoking) and disease (hypertension and diabetes) data were gathered from the TWB questionnaires. Using colorimetric assays (Hitachi LST008, Automatic Clinical Chemistry Analyzer, Hitachi, Naka, Japan), individual lipid levels, including HDL cholesterol, triglycerides (TG), and non-HDL cholesterol were measured from blood samples collected at TWB assessment centers.

In this study, exercise habits were divided into three categories: no exercise, aerobic exercise, and resistance exercise. Subjects who regularly exercised for at least 30 min a day, three times a week, were considered regular exercisers. Ball games and weight training were included in the study as forms of resistance exercise. Aerobic exercise included Chinese martial arts, brisk walking, Taijiquan, hiking, jogging, basketball, rope jumping, gymnastics, yoga, qigong, swimming, biking, table tennis, tennis, soccer, badminton, aerobic dance, hula hooping, golf, and ballroom dance.

### 2.3 Variant rs7412 of the APOE gene

Our study focused on variant rs7412 of the *apolipoprotein E* gene, which was chosen based on literature searches. The reason for analyzing the variant was that it represents one of those variants that are known to affect plasma lipid levels as mentioned above. To our knowledge, only a few studies have used TWB to examine this variant in the context of TG, Alzheimer’s, hypertension, C-reactive protein (CRP), and diabetic neuropathy.

### 2.4 Data analysis

The Chi-square test and Student’s t-test were used to examine differences in the distribution of study variables (categorical and continuous variables) based on CC, CT + TT genotypes of the variant rs7412. To examine the association between exercise, APOE rs7412, and HDL cholesterol, we used multiple linear regression models that controlled for age, sex, cigarette smoking, alcohol consumption, body mass index (BMI), triglycerides (TG), non-HDL, diabetes, hypertension, and serum glutamate pyruvate transaminase (SGPT), serum glutamic oxaloacetic transaminase (SGOT), and gamma-glutamyl transferase (GGT). We tested for a possible interaction between APOE rs7412 and exercise using a multiple linear regression model, followed by stratified analyses of these variables. Covariates included in the interaction model included age, sex, cigarette smoking, alcohol consumption, BMI, TG, non-HDL, diabetes, and hypertension, SGPT, SGOT and GGT. The significance levels were based on a *p*-value less than 0.05. Besides Plink version 2.0, SAS 9.4 software (SAS Institute, Cary, NC, United States) was used for data analysis.

## 3 Results

A total of 57,638 non-hyperlipidemic subjects (39,144 women and 18,494 men) were included in this study (Table 1). The mean (SE) HDL cholesterol level was 54.794 (0.060) mg/dL in rs7412-CC subjects and 56.549 (0.155) mg/dL in rs7412-CC + TT subjects. *APOE* rs7412-CC subjects made up 49,395 of the sample, while *APOE* rs7412-CT+TT subjects made up 8,251. There were two types of exercise in the multiple regression model: aerobic and resistance (Table 2). In comparison with the no exercise group, both aerobic and resistance exercise were associated with increased HDL cholesterol: The β-coefficient (95% CI) was 1.112 (0.903–1.322) and 2.530 (2.093–2.966), respectively. In comparison with the rs7412-CC genotype, the β-coefficient was 2.589 (95% CI 2.329–2.848) among those with the CT + TT genotype. With normal weight representing the reference group, the β-coefficients (95% CI) were 6.128 (5.634–6.621), −4.323 (−4.547–−4.100), −6.519 (−6.777 to −6.261) for the underweight, overweight, and obese categories, respectively. Men compared to women had significantly lower levels of HDL cholesterol (β = −7.066, 95% CI -7.295 to −6.838).

**TABLE 1:** Demographic characteristics of all subjects based on the APOE rs7412 genotypes.

Variables	APOE rs7412 CC (n = 49,387, %)	APOE rs7412 CT + TT (n = 8,251, %)	*p*-value
HDL (mg/dL)±SE	54.794 ± 0.060	56.549 ± 0.155	<0.001
Exercise habit			0.532
No exercise	32,880 (66.58)	5,542 (67.17)	
Aerobic exercise	14237 (28.83)	2344 (28.41)	
Resistance exercise	2270 (4.60)	365 (4.42)	
Sex			0.380
Women	33,506 (67.84)	5,638 (68.33)	
Men	15,881 (32.16)	2613 (31.67)	
Age (mean ± SE)	48.778 ± 0.047	48.788 ± 0.116	0.937
Cigarette smoking			0.725
Never	40181 (81.36)	6735 (81.63)	
Former	4653 (9.42)	778 (9.43)	
Current	4553 (9.22)	738 (8.94)	
Alcohol consumption			0.259
Never	45,500 (92.13)	7592 (92.01)	
Former	1163 (2.35)	177 (2.15)	
Current	2724 (5.52)	482 (5.84)	
BMI			0.478
Underweight (<18.5)	1762 (3.57)	270 (3.27)	
Normal (18.5≤BMI<24)	24,868 (50.35)	4137 (50.14)	
Overweight (24≤BMI<27)	12,961 (26.24)	2172 (26.32)	
Obese (BMI≥27)	9796 (19.84)	1672 (20.26)	
TG (mg/dL)	110.90 ± 0.372	118.30 ± 0.963	<0.001
Non-HDL (mg/dL)	142.70 ± 0.152	126.80 ± 0.380	<0.001
Diabetes			0.640
No	47,589 (96.36)	7942 (96.25)	
Yes	1798 (3.64)	309 (3.75)	
Hypertension			0.550
No	44,796 (90.70)	7501 (90.91)	
Yes	4591 (9.30)	750 (9.09)	
SGPT	23.109 ± 0.094	23.402 ± 0.216	0.213
SGOT	24.535 ± 0.056	24.756 ± 0.127	0.111
GGT	23.272 ± 0.126	23.624 ± 0.353	0.349

Abbreviations: SE, standard error; TG, triglycerides; BMI, body mass index, CC, CT, and TT = APOE, rs7412 genotypes, SGPT, serum glutamate pyruvate transaminase; SGOT, serum glutamic oxaloacetic transaminase; GGT, gamma-glutamyl transferase.

The Chi-Square test was used for categorical data and the Student’s t-test for continuous data.

**TABLE 2:** HDL cholesterol levels determined by multiple linear regression analysis.

Variables	β-coefficient	95% C.I.	*p*-value
Exercise habit (ref.: no exercise)			
Aerobic exercise	1.112	0.903–1.322	<0.001
Resistance exercise	2.530	2.093–2.966	<0.001
APOE rs7412 (ref.: CC)			
CT + TT	2.589	2.329–2.848	<0.001
Sex (ref.: Women)			
Men	−7.066	−7.295–−6.838	<0.001
Age	0.036	0.026–0.045	<0.001
Cigarette smoking (ref.: Never)			
Former	−0.121	−0.458–0.217	0.484
Current	−1.900	−2.244–−1.557	<0.001
Alcohol consumption (ref.: Never)			
Former	−0.502	−1.110–0.106	0.106
Current	4.690	4.273–5.108	<0.001
BMI(ref.: Normal)			
Underweight	6.128	5.634–6.621	<0.001
Overweight	−4.323	−4.547–−4.100	<0.001
Obese	−6.519	−6.777–−6.261	<0.001
TG	−0.056	−0.057–−0.055	<0.001
Non-HDL	0.027	0.024–0.030	<0.001
Diabetes (ref.: No)			
Yes	−2.123	−2.613–−1.633	<0.001
Hypertension (ref.: No)			
Yes	−1.490	−1.816–−1.164	<0.001
SGPT	−0.075	−0.084–−0.066	<0.001
SGOT	0.113	0.099–0.127	<0.001
GGT	0.026	0.022–0.029	<0.001

Abbreviation: CI, confidence interval; TG, triglycerides; BMI, body mass index, CC, CT, and TT = APOE, rs7412 genotypes, SGPT, serum glutamate pyruvate transaminase; SGOT, serum glutamic oxaloacetic transaminase; GGT, gamma-glutamyl transferase.

In addition, there was evidence of an interaction between APOE rs7412 and exercise (*p* for interaction = 0.049). Further stratification by the SNP genotypes (Table 3) showed β-values (95% CI) of 1.111 (0.887–1.335) and 2.722 (2.256–3.189) for aerobic exercise and resistance exercise among CC genotype individuals, and 1.112 (0.533–1.691) and 1.374 (0.150–2.598), respectively among those with the CT + TT genotype.

**TABLE 3:** HDL cholesterol levels by APOE rs7412 genotypes based on multiple linear regression.

Variables	APOE rs7412 = CC				APOE rs7412 = CT + TT			
	N	β -coefficient	95%C.I.	*p*-value	N	β -coefficient	95%C.I.	*p*-value	
Exercise habit (ref.: no exercise)	32,880				5,542				
Aerobic exercise	14,237	1.111	0.887–1.335	<0.001	2344	1.112	0.533–1.691	<0.001	
Resistance exercise	2270	2.722	2.256–3.189	<0.001	365	1.374	0.150–2.598	0.028	
Sex (ref.: Women)	33,606				5,638				
Men	15,881	−6.947	−7.191–−6.702	<0.001	2613	−7.782	−8.413–−7.150	<0.001	

Adjusted for age, cigarette smoking, alcohol consumption, BMI, TG, non-HDL, diabetes, and hypertension, SGPT, SGOT, and GGT.

Abbreviation: CI, confidence interval; TG, triglycerides; BMI, body mass index, CC, CT, and TT = APOE, rs7412 genotypes.

In a multiple regression model combining rs7412 genotypes and exercise types (Table 4) and using the CC genotype and no exercise group as a reference group, the β-coefficients (95% CI) were 1.135 (0.911–1.359) for the CC genotype and aerobic exercise group, 2.753 (2.283–3.222) for the CC genotype and resistance exercise group, 2.705 (2.390–3.020) for the CT + TT genotype and no exercise group, 3.682 (3.218–4.146) for the CT + TT genotype and aerobic exercise group, and 3.855 (2.727–4.982) for the CT + TT genotype and resistance exercise group, respectively.

**TABLE 4:** HDL cholesterol levels based on a combination of exercise modalities and APOE rs7412 genotypes.

Variables	N	β	95%C.I.	*p*-value
APOE rs7412, exercise habit (ref.: CC, no exercise)	32,880			
CC, aerobic exercise	14,237	1.135	0.911–1.359	<0.001
CC, resistance exercise	2270	2.753	2.283–3.222	<0.001
CT + TT, no exercise	5,542	2.705	2.390–3.020	<0.001
CT + TT, aerobic exercise	2344	3.682	3.218–4.146	<0.001
CT + TT, resistance exercise	365	3.855	2.727–4.982	<0.001

Adjusted for age, sex, cigarette smoking, alcohol consumption, BMI, TG, non-HDL, diabetes, hypertension, SGPT, SGOT, and GGT.

## 4 Discussion

The apolipoprotein, *APOE* plays an instrumental role in regulating HDL cholesterol levels. It has also been shown in previous research that the function and composition of HDL are influenced by lifestyle factors during midlife (Nasr et al., 2022). Researchers have also indicated that exercise-induced changes in HDL cholesterol are modified by genetic polymorphisms (Blazek et al., 2013). Using data from Taiwan Biobank subjects aged 30–70 years who were recruited between 2008 and 2019, we examined whether the association between HDL cholesterol and exercise is related to the APOE variant. To test this hypothesis, specifically, we used the variant *APOE* rs7412, well-known for its association with HDL cholesterol levels. As part of our analysis, we looked at exercise categories such as aerobics and resistance exercises, and SNP genotypes, CT + TT and CC, respectively. We found that self-reported resistance and aerobic exercise were associated with higher HDL cholesterol but the increase was more pronounced with resistance exercise. The trend was maintained regardless of SNP genotypes added to the analysis models. For instance, CT + TT individuals who engaged in resistance exercise demonstrated elevated HDL cholesterol levels [β = 4.032 (95% CI 3.249 to 4.181)], surpassing those who engaged in aerobic exercise [β = 3.715 (95% CI 3.249 to 4.181)], in comparison to individuals with the CC genotype, as determined by multivariate regression analysis. Despite these, further research is needed to explore the possible mechanisms involved.

Researchers have previously proposed that a reduction in cardiovascular disease risk may also be associated with exercise-induced increases in HDL function (Stanton et al., 2022). Our study suggests that these increases may occur more in *APOE* rs7412-CT+TT individuals with the *APOE* gene in Taiwan. It has been proposed that *apolipoprotein E* together with exercise training can greatly affect lipoprotein metabolism (Thompson et al., 2004). Currently, genetic variations linked to HDL cholesterol and exercise training have not been extensively explored, especially in Taiwan. Consequently, the results of the current study could serve as a reference point for future epidemiological studies.

HDL cholesterol levels can be improved in later life by keeping a healthier body mass index, according to a previous study (Nasr et al., 2022). In the current study, we found significant associations between HDL cholesterol and BMI. That is, compared with normal BMI, lower BMI (underweight category) was associated with a greater increase in HDL cholesterol, whereas higher BMI (overweight and obese categories) was associated with lower HDL cholesterol.

We found that several factors were inversely related to HDL cholesterol, including smoking, diabetes, and hypertension. Earlier research indicated that smoking adversely affects both HDL quantity and function, thereby increasing cardiovascular disease risk (He et al., 2013). To our knowledge, positive and negative findings have been reported regarding the relationship between alcohol consumption and HDL cholesterol. There has been an indication, however, that the type of alcohol consumed affects HDL cholesterol levels (Huang et al., 2017).

Overall, the study contributes to our understanding of the interplay between genetics, exercise, and HDL cholesterol levels, providing valuable insights that can inform future research, clinical practice, and public health strategies aimed at promoting cardiovascular health. It is important, however, to acknowledge the limitations of our study. Data used in the current study may not have sufficient scope or outcomes to accurately explain the relationship of HDL cholesterol with genetic and lifestyle factors. Given that exercise intensity is emerging as the most potent prescriptive factor in disease modification and improving metabolic outcomes, the absence of this crucial descriptor does make it hard to appropriately determine the true relationship between physical exercise and HDL cholesterol. Lastly, our study did not examine the possible impact of diet on HDL cholesterol levels.

## 5 Conclusion

This study observed that the association between HDL cholesterol and APOE rs7412 varied according to the type of exercise performed. Specifically, individuals with the T-allele (CT and TT) exhibited a more notable increase in HDL cholesterol compared to those with the C-allele (CC), particularly in the context of engaging in resistance training. These results have implications for clinical practice and public health initiatives. Promoting regular exercise, especially resistance exercise, may be a useful strategy for improving HDL cholesterol levels and reducing the risk of cardiovascular diseases in the general population. Genetic screening for APOE rs7412 genotype could be considered to identify individuals who may benefit the most from specific exercise interventions.

## Data Availability

The data analyzed in this study is subject to the following licenses/restrictions: data used in this study are held by the Taiwan Biobank (https://www.twbiobank.org.tw/new_web/) and are available from the authors upon reasonable request or directly from the Taiwan Biobank (biobank@gate.sinica.edu.tw). requests to access these datasets should be directed to https://www.twbiobank.org.tw/new_web/.
